# Dietary Supplementation with *Bacillus licheniformis* and *Bacillus subtilis* Modulates Immunity, Serum Metabolome, and Intestinal Homeostasis in Cats

**DOI:** 10.3390/ani15202971

**Published:** 2025-10-14

**Authors:** Meiting Zhang, Haocheng Xu, Tianfeng Zhang, Jia Kang, Zhihao Xu, Peng Wu, Yu Niu, Yonghao Shi, Yifan Zhong, Caimei Yang

**Affiliations:** College of Animal Science and Technology, College of Veterinary Medicine, Zhejiang Agricultural and Forestry University, Hangzhou 311300, China; mittyours@163.com (M.Z.); haochengxu03@foxmail.com (H.X.); zhangtf1203@163.com (T.Z.); hnkjhh@163.com (J.K.); 18057686089@163.com (Z.X.); w19939905069@163.com (P.W.); niuyu0227@126.com (Y.N.); m19157736914@163.com (Y.S.)

**Keywords:** *Bacillus licheniformis*, *Bacillus subtilis*, immunity, serum metabolome, gut health, cat

## Abstract

In recent years, as the number of cats has increased, the intestinal health of cats has receied increasing amounts of attention. Probiotics have positive effects on maintaining intestinal homeostasis. This study was conducted to investigate the effects of probiotics *Bacillus licheniformis* (*B. licheniformis*) and *Bacillus subtilis* (*B. subtilis*) on cat immunity, inflammation, antioxidants, intestinal barrier and microbiota, and serum metabolites. Thirty-six cats (over one year old, 3.48 ± 0.71 kg) were randomly divided into 3 groups and fed with a basal diet (CON group), a basal diet + *B. licheniformis* (BL group), and a basal diet + *B. subtilis* (BS Group). The experiment lasted 35 days. Fecal scoring indicates that *B. licheniformis* and *B. subtilis* can improve fecal scores. Serum analysis indicated that the addition of both substances increased levels of IgA, IgM, T-AOC, and SOD, while reducing levels of the pro-inflammatory factor TNF-α. Moreover, 16S rRNA gene sequencing revealed that *B. licheniformis* and *B. subtilis* altered the fecal microbiota composition, characterized by the elevated abundance of *Bacillus*. Adding *B. licheniformis* to the diet increased the level of *Faecalibacterium* and decreased the level of *Mogibacterium*. Serum metabolomics revealed that levels of L-Glycine and Sn-Glycero-3-phosphocholine exhibited marked elevation in both the BL and BS groups, respectively. Furthermore, the study demonstrated that differential metabolites in the BL group were mainly enriched in amino acid metabolism pathways, while those in the BS group were chiefly concentrated in lipid metabolism pathways. However, this study acknowledges the limitations of its exclusive use of Ragdoll cats and its 35-day intervention period. It highlights the need for future research involving diverse breeds and longer durations. Overall, the data highlight *B. licheniformis* and *B. subtilis* as cat nutritional supplements that improve immunity and maintain intestinal health.

## Simple Summary

The health of domestic cats, particularly gastrointestinal well-being, is garnering growing scientific interest, with probiotics recognized for their crucial role in sustaining gut homeostasis. This study investigates the impact of probiotics (*Bacillus licheniformis*, *Bacillus subtilis*) on feline immunity, serum metabolomics, and intestinal homeostasis. The results suggest that *Bacillus licheniformis* and *Bacillus subtilis* improve fecal quality, mitigate inflammatory responses, enhance immune function, and strengthen the intestinal barrier. Furthermore, *Bacillus licheniformis* modulates amino acid metabolic pathways, while *Bacillus subtilis* regulates lipid metabolic pathways. Together, these findings suggest that both strains hold promfecalas feed additives for improving feline gut health.

## 1. Introduction

As dogs and cats occupy an increasingly important position in households, their health has garnered significant attention. Due to the rising ownership rate of cats, feline health has received increasing attention in recent years, driven by the rising number of cat owners. The gut microbiota, as an important part of the intestine, takes part in the digestion, absorption, and metabolism of nutrients [1]. A healthy and stable intestinal microbiota can resist inflammation and maintain the health of the host [2]. As a primary strategy for modulating gut microbiota, probiotics maintain the microecological homeostasis of the intestine by altering the structure of gut microbiota, thereby preserving the physical health of felines [3,4]. A study has found that chronic intestinal inflammation associated with feline inflammatory bowel disease (IBD) involves a sophisticated interplay between the mucosal immune system and gut microbiota in a host with genetic susceptibility [5]. A robust gut microbiome exhibits dual pro- and anti-inflammatory properties, maintaining homeostatic equilibrium to mitigate pathological hyperinflammation, while preserving immune responsiveness to infectious challenges [6]. The tripartite interplay governing probiotic–host cell–microbiota dynamics critically modulates host physiological homeostasis [7].

Probiotics, recognized as bioactive microorganisms conferring host health benefits, have been used as a functional food and dietary supplements, with emerging applications in canine and feline health management. Probiotics play a significant role in improving intestinal health primarily by regulating the balance of the gut microbiota, strengthening the intestinal barrier, and modulating immune responses [8]. Probiotics limit the colonization of pathogenic bacteria by competing for intestinal binding sites and nutritional resources, while concurrently enhancing the intestinal barrier function and reducing epithelial permeability [9]. *B. licheniformis* can form spores; its multiple benefits mainly focus on the gastrointestinal and immune systems. *B. licheniformis* alleviates the subhealth state through recovering the gut microbiota balance and reducing inflammation [10]. *B. licheniformis* BCG facilitated the digestion and absorption of nutrients in broilers, strengthened the intestinal physical barrier, and alleviated intestinal inflammation by altering microbial diversity and optimizing the microbiota structure. *B. subtilis* is environmentally stress-resistant due to its ability to form spores. It also synthesizes bioactive compounds—such as vitamins, enzymes, and short-chain fatty acids—that confer benefits to the host [11]. *B. subtilis* DE-CA9^TM^ intervention significantly ameliorated key serum oxidative stress markers, including advanced oxidation protein products (AOPP), diacron-reactive oxygen metabolites (d-ROMS), and thiobarbituric acid reactive substances (TBARS) [12]. Dietary supplementation with 0.01% *B. subtilis* C3102 enhanced canine intestinal health and fecal quality, evidenced by reduced ammonia concentration and improved fecal consistency [13]. Previous research conducted in our laboratory has demonstrated that *B. licheniformis* HJ0135 enhances immune responses, improves growth performance, and mitigates lipopolysaccharide (LPS)-induced inflammatory damage in weaned piglets [14]. However, research into *B. Licheniformis* in cats is still in its early stages.

Although *B. licheniformis* and *B. subtilis* have demonstrated beneficial effects on intestinal health, their applications and beneficial effects in feline species remain limited. Moreover, studies investigating the metabolomic effects in felines and the microbiota-metabolite interactions remain scarce. This study sought to elucidate how *B. licheniformis* and *B. subtilis* affect the feline gut microbiota and metabolome via multi-omics techniques, thereby demonstrating their potential functions in regulating intestinal health.

## 2. Materials and Methods

### 2.1. Animals and Experimental Treatments

**Figure 1:**
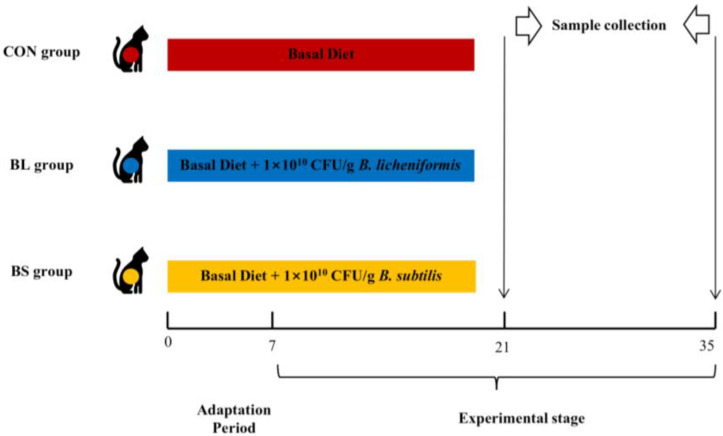
The animal experimental protocol. CON group: fed with a basal diet, BL group: fed with a basal diet and 1 × 10^10^ CFU/g *B. licheniformis*, BS group: fed with a basal diet and 1 × 10^10^ CFU/g *B. subtilis*.

Thirty-six healthy adult cats (Ragdoll cats) aged one year or older, (3.48 ± 0.71 kg) were randomly divided into three groups (*n* = 12 per group), and all cats were housed individually throughout the study. The feeding experiments were conducted for 35 days, comprising a 7-day adaptation period followed by a 28-day formal. The groups were assigned as follows: control group (CON), fed with the basal diet; *B. licheniformis* group (BL), provided with the basal diet supplemented with 1 × 10^10^ CFU/g of *B. licheniformis*; *B. subtilis* group (BS), served the basal diet supplemented with 1 × 10^10^ CFU/g of *B. subtilis.* Figure 1 illustrates the experimental design. The probiotics utilized in this study were supplied by Zhejiang Huijia Bio-technology Co., Ltd. (Huzhou, China). Before the trial, all cats received comprehensive immunization and deworming. All cats were free from medications known to affect gut microbiota for at least one month preceding the trial. Feces were collected twice daily. Every cat was supplied with 70 g of food once daily and had free access to clean drinking water at all times. The 28-day research phase was started after one week of acclimation. During the trial, body weight was assessed on days 1, 7, 14, 21, 28, and feed intake per day was noted to calculate the average daily feed intake (ADFI).

### 2.2. Sample Collection

A volume of 2 mL of blood was taken from the cephalic vein located in the forelimb of six randomly selected cats from each group on the 14th and 28th day. The serum was gathered by centrifugation at 3000 rpm for 15 min at 4 °C and preserved at −20 °C until further testing. Simultaneously, fresh feces were collected from six randomly selected cats from each group on day 28 of the experiment using sterilized forceps and transferred into a cryopreservation tube, then kept at −80 °C.

The fecal scoring was evaluated according to Table 1 on days 1–7, 14, and 28.

### 2.3. Serum Biochemical Analysis

Serum antioxidant indices, including superoxide dismutase (SOD), total antioxidant capacity (T-AOC), glutathione peroxidase (GSH-Px), catalase (CAT), D-lactic acid (DLA), and diamine oxidase (DAO) were detected using kits commercially available from the Ao Qing Biotechnology Co., Ltd. (Nanjing, Jiangsu, China).

### 2.4. Immune Cytokine Analysis

Serum concentrations of tumor necrosis factor-α (TNF-α), interferon-γ (IFN-γ), interleukin-2 (IL-2), interleukin-4 (IL-4), immunoglobulin (Ig)G, IgA, and IgM were quantified using enzyme-linked immunosorbent assay (ELISA). All assay kits were acquired from Ao Qing Biotechnology Co., Ltd. (Nanjing, Jiangsu, China).

### 2.5. Fecal Microbiome Analysis

Genomic DNA was extracted from fecal samples using the E.Z.N.A.^®^ Stool DNA Kit (Omega Bio-tek, Norcross, GA, USA). The V3–V4 hypervariable regions of the bacterial 16S rRNA gene were subsequently amplified by polymerase chain reaction (PCR) with the universal primers 338F (5′–ACTCCTACGGGAGGCAGCA–3′) and 806R (5′–GGACTACHVGGGTWTCTAAT–3′). PCR products were purified using the AxyPrep DNA Gel Extraction Kit and subjected to paired-end sequencing on an Illumina MiSeq PE300 platform (Illumina, San Diego, CA, USA). Quantification was performed with a Quantus Fluorometer (Promega, Madison, WI, USA). Raw sequencing data were demultiplexed and quality-filtered with Fastp (v0.20.0). Raw sequencing data were processed in QIIME 2 (version 2020.8; (https://qiime2.org, accessed on 6 March 2024)).

To perform taxonomic annotation of ASVs, the Naive Bayes was applied, and the abundance of each classified ASV was quantified across all samples. Alpha diversity was assessed via the Shannon and Chao indices to quantify both species richness and evenness within each sample. Principal Coordinate Analysis (PCoA) based on Bray–Curtis illustrated the similarity of communities. Bacterial abundance was calculated as a percentage of abundance at the phylum and genus taxonomic levels. The Kruskal–Wallis rank sum test was used to evaluate the significant differences in species abundance, and the species information with significant differences among multiple groups was obtained.

### 2.6. Analysis of Serum Metabolomics

Metabolite extraction was conducted by combining 100 μL of serum with acetonitrile/methanol (1:1, *v*/*v*). The samples were vortexed for 30 s and sonicated at low temperature for 30 min, then incubated at −20 °C for 30 min to induce protein precipitation. After centrifugation at 13,000 rpm and 4 °C for 15 min, the supernatant was removed and blown dry using nitrogen gas. The residue was redissolved in 100 μL of a 1:1 acetonitrile-water solution, sonicated at 4 °C for 5 min, and centrifuged once more at 13,000 rpm and 4 °C for 10 min. The resulting supernatant was transferred to injection vials for LC-MS/MS analysis, which was performed by Majorbio Bio-Pharm Technology Co., Ltd. (Shanghai, China) using a UHPLC-Orbitrap Exploris 240 system fitted with an ACQUITY UPLC HSS T3 column (100 mm × 2.1 mm, 1.8 μm; Waters, Milford, MA, USA).

Orthogonal projections to latent structures-discriminant analysis (OPLS-DA) and permutation tests for model validation were implemented using the R package “ropls” (version 1.6.2). Differential metabolites were chosen according to the thresholds of a variable importance in projection (VIP) score exceeding 2.0 and a *p*-value less than 0.05 from Student’s *t*-test. MetaboAnalyst 5.0 was utilized for pathway enrichment analysis, with significantly changed Kyoto Encyclopedia of Genes and Genomes (KEGG) pathways detected via the hypergeometric test and adjusted for multiple comparisons (FDR < 0.05).

### 2.7. Statistical Analysis

SPSS software (version 27.0; SPSS Inc., Chicago, IL, USA) was utilized for the analysis of experimental data, with statistical significance set at *p* < 0.05. GraphPad Prism software (version 9; Dotmatics, Boston, MA, USA) was employed to create the graphics. Values of *p* < 0.05 (*) and *p* < 0.01 (**) were considered statistically significant and extremely significant, respectively. When comparing groups under the same indicator, significant differences are indicated by entries labeled with different letters (a, b, c) (*p* < 0.05).

## 3. Results

### 3.1. Growth Performance

Table 2 illustrates the effects of probiotics on feline body weights and ADFI throughout the course of the experiment. No significant difference was observed in the body weight and ADFI.

### 3.2. Fecal Scores

Fecal scoring is presented in Table 3. On days 3, 5, 7, 14, and 21, the cats in the BS group had higher fecal scores than those in the CON group (*p* < 0.05); meanwhile, the fecal scores of the BL group on days 4, 5 had a higher rating than the CON group.

### 3.3. Immunoglobulin Parameters

**Figure 2:**
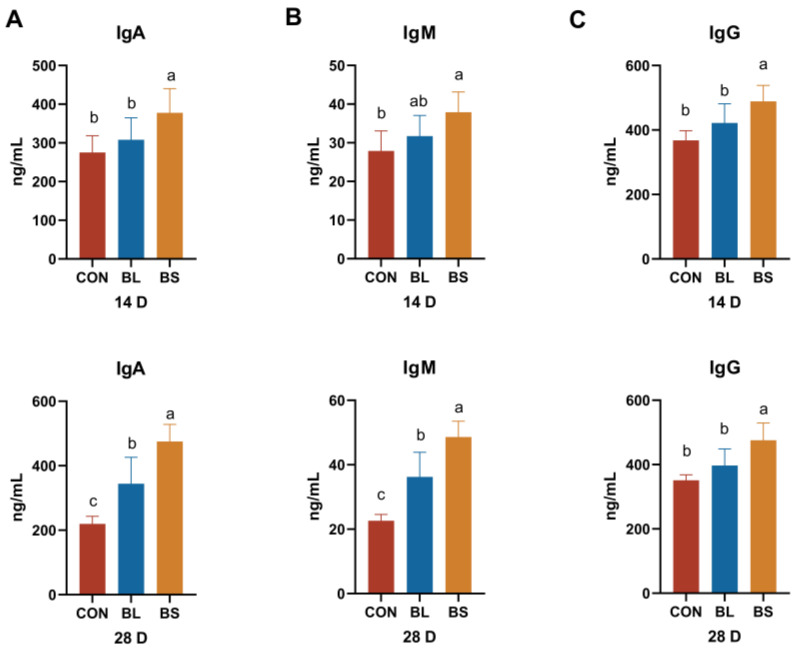
Effects of BL and BS on immunoglobulin parameters in cats. (**A**) IgA content; (**B**) IgM content; (**C**) IgG content. CON: fed with basic diet; BL: fed with the basal diet and 1 × 10^10^ CFU/g *B. licheniformis*; BS: fed with the basal diet and 1 × 10^10^ CFU/g *B. subtilis.* Data are expressed as mean ± SD (*n* = 6 per group). a, b, c: means within the same row that have different superscripts are significantly different (*p* < 0.05).

BS treatment significantly increased the level of the IgA, IgM, and IgG in the blood, as depicted on days 14 and 28 in Figure 2 (*p* < 0.05). The IgA and IgM concentrations in the BL group differed significantly from those in the CON group by day 28 (*p* < 0.05). However, the IgG of the BL group had no significant difference compared with the CON group.

### 3.4. Antioxidant Parameters

**Figure 3:**
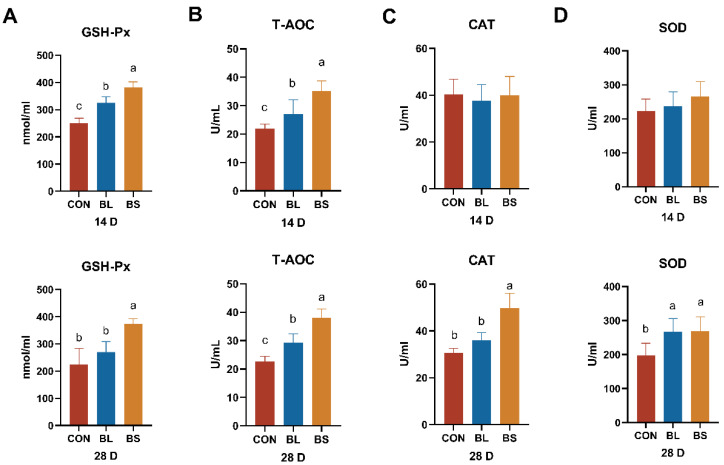
Effects of the BL and BS on serum antioxidant indexes in cats. (**A**) GSH-Px content; (**B**) T-AOC content; (**C**) CAT content; (**D**) SOD content. CON: fed with basic diet; BL: fed with the basal diet and 1 × 10^10^ CFU/g *B. licheniformis*; BS: fed with the basal diet and 1 × 10^10^ CFU/g *B. subtilis.* Data are expressed as mean ± SD (*n* = 6 per group). a, b, c: means within the same row that have different superscripts are significantly different (*p* < 0.05).

The effects of probiotics on antioxidant markers in feline serum were evaluated by quantifying the concentrations of CAT, SOD, GSH-Px, and T-AOC, and the relevant results are presented in Figure 3. Relative to the CON group, the BL and BS groups raised the level of GSH-Px and T-AOC on day 14 (*p* < 0.05). The BS group exhibited higher GSH-Px, T-AOC, CAT, and SOD on day 28 (*p* < 0.05). Additionally, the BL group had significantly elevated serum T-AOC and SOD levels on day 28 relative to the CON group. However, there were no significant differences in serum GSH-Px and CAT values in cats on day 28.

### 3.5. Inflammatory Factors

**Figure 4:**
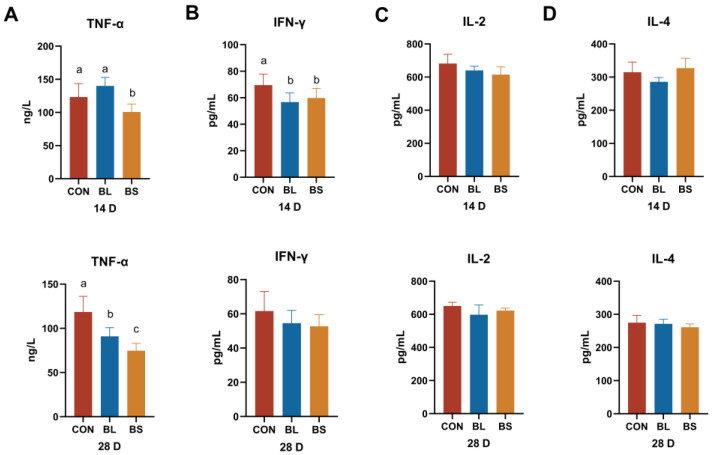
Effects of the BL and BS on plasma inflammatory parameters in cats. (**A**) TNF-α content; (**B**) IFN-γ content; (**C**) IL-2 content; (**D**) IL-4 content. CON: fed with basic diet; BL: fed with the basal diet and 1 × 10^10^ CFU/g *B. licheniformis*; BS: fed with the basal diet and 1 × 10^10^ CFU/g *B. subtilis.* Data are expressed as mean ± SD (*n* = 6 per group). a, b, c: means within the same row that have different superscripts are significantly different (*p* < 0.05).

The impacts of the BL group and BS group on the serum cytokines are illustrated in Figure 4. Relative to the control group, the BS group exhibited a significant reduction in TNF-α and IFN-γ levels on day 14 (*p* < 0.05). In addition, IFN-γ levels in the BL group were significantly lower than those in the CON group on day 14 (*p* < 0.05). On day 28, TNF-α content in both the BL and BS groups was significantly lower than that in the CON group (*p* < 0.05).

### 3.6. Gut Barrier Function Parameters

**Figure 5:**
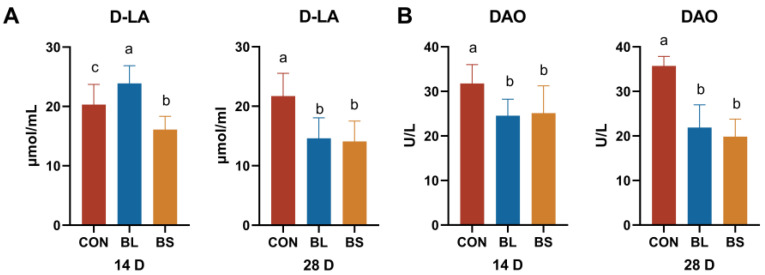
Effects of BL and BS on plasma intestinal barrier function parameters in cats. (**A**) D-LA content; (**B**) DAO content. CON: fed with basic diet; BL: fed with the basal diet and 1 × 10^10^ CFU/g *B. licheniformis*; BS: fed with the basal diet and 1 × 10^10^ CFU/g *B. subtilis.* Data are expressed as mean ± SD (*n* = 6 per group). a, b, c: means within the same row that have different superscripts are significantly different (*p* < 0.05).

Figure 5 depicts the serum levels of D-LA and DAO. In contrast to the CON group, the BL and BS group markedly reduced the serum concentrations of D-LA and DAO on day 28 (*p* < 0.05). Moreover, DAO levels had decreased in both the BS and BL groups, but DLA levels had decreased only in the BS group on day 14 (*p* < 0.05).

### 3.7. Fecal Microbiota Composition

**Figure 6:**
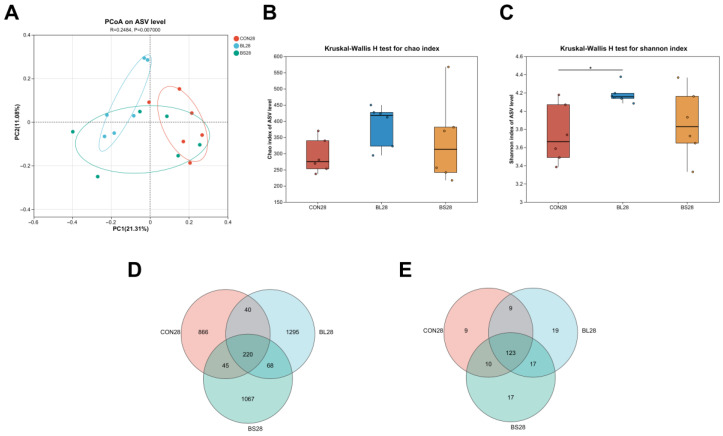
Effect of BL and BS on the composition of fecal microbiota in cats. (**A**) PCoA analysis; (**B**) Chao index; (**C**) Shannon index; (**D**) The Venn diagram summarizes the abundance of common and unique ASVs within the fecal microbiota. (**E**) The Venn diagram summarizes the abundance of common and unique genus within the fecal microbiota. CON: fed with basic diet; BL: fed with the basal diet and 1 × 10^10^ CFU/g *B. licheniformis*; BS: fed with the basal diet and 1 × 10^10^ CFU/g *B. subtilis.* * *p* ≤ 0.05. Data are expressed as mean ± SD (*n* = 6 per group).

Figure 6 presents the results of cat fecal microbiota diversity analysed using 16S rRNA sequencing technology. PCoA revealed distinct clustering of microbial communities according to group assignment, with clear separation observed among all three groups. (Figure 6A, *p* = 0.007). According to the Shannon index, the diversity of the CON group differed significantly from that of the BL group, but the Chao index revealed that there were statistically insignificant differences among the groups. (Figure 6B,C; *p* > 0.05). In order to characterize the taxonomic overlap across groups, an analysis was conducted of shared and unique taxa at both the amplicon sequence variants (ASVs) and genus levels. Across fecal samples from the three experimental groups, 3601 ASVs were identified in total at the ASV level. Among these, 220 ASVs were shared by all groups, while 1295 and 1067 ASVs were uniquely identified in the BL and BS groups, respectively. (Figure 6D). At the genus level, the common microbial genera of the CON, BL and BS groups were 123, and the unique genera of the three groups were 9, 19, and 17, respectively (Figure 6E).

**Figure 7:**
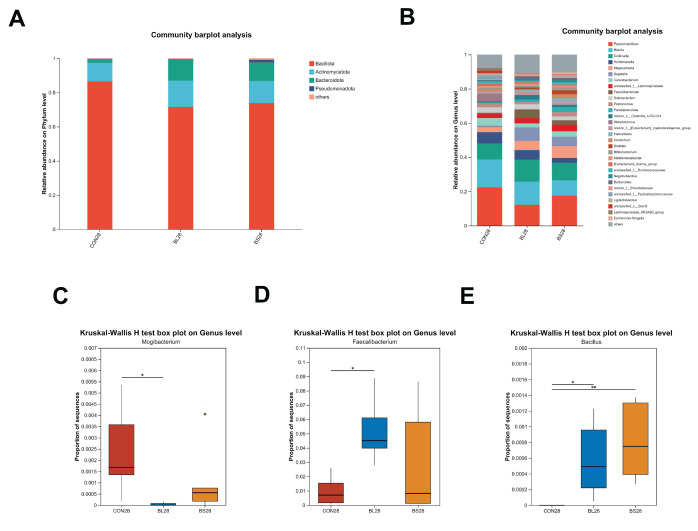
Effect of BL and BS on differences at phylum and genus levels in cats. (**A**) Bar graph of microbial composition at the phylum level. (**B**) Bar graph of microbial composition at the genus level. (**C**–**E**) Genus-level analysis of relative abundance of microbial communities in fecal contents CON: fed with basic diet; BL: fed with the basal diet and 1 × 10^10^ CFU/g *B. licheniformis*; BS: fed with the basal diet and 1 × 10^10^ CFU/g *B. subtilis.* * *p* ≤ 0.05, ** *p* ≤ 0.01. Data are expressed as mean ± SD (*n* = 6 per group).

Figure 7A,B illustrate the relative abundance of gut microbiota in cats, categorized by phylum and genus, respectively. At the phylum level, Bacillota represented the most abundant bacterial taxon in all groups, with initial relative abundances of 86.54% (CON group), 71.56% (BL group), and 73,78% (BS group) (Figure 7A). At the genus level, *Peptoclostridium*, *Blautia*, *Collinsella*, *Holdemanella*, and *Megasphaera* are the main dominant bacterial communities (Figure 7B). In addition, at the genus level, the relative abundance of *Faecalibacterium* and *Bacillus* was significantly increased in the BL group (Figure 7D; *p* < 0.05), while the relative abundance of *Bacillus* was significantly increased in the BS group (Figure 7E; *p* < 0.01). The relative abundance of *Mogibacterium* decreased significantly compared to the CON group after 28 days of feeding with BL (Figure 7C; *p* < 0.05).

### 3.8. Serum Metabolomics

**Figure 8:**
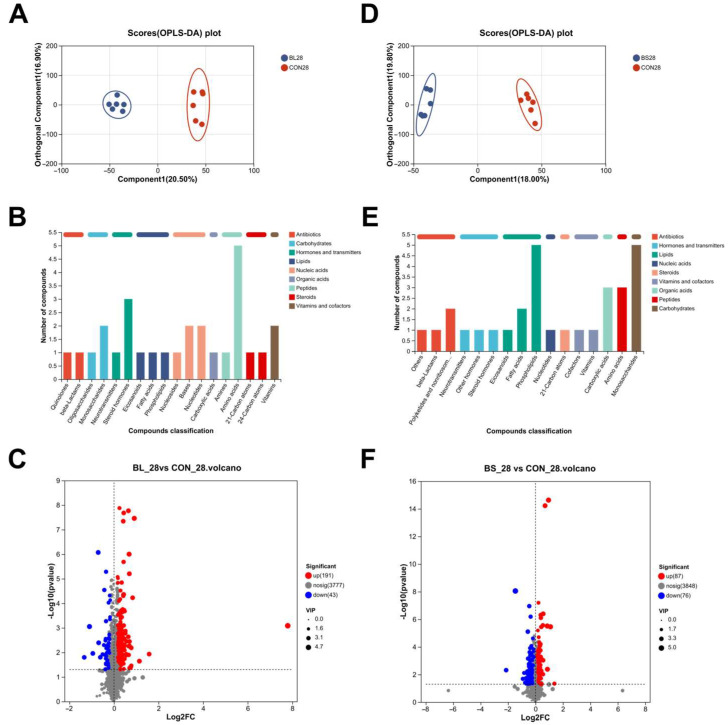
Effect of BL and BS on the serum metabolomics in cats. (**A**) OPLS-DA score plot for group BL; (**B**) KEGG compound classification diagram for BL group; (**C**) Volcano plot analysis of differentially expressed metabolites in BL group serum; (**D**): OPLS-DA score plot for group BS; (**E**) KEGG compound classification diagram for BS group; (**F**) Volcano plot analysis of differentially expressed metabolites in BS group serum. CON: fed with basic diet; BL: fed with the basal diet and 1 × 10^10^ CFU/g *B. licheniformis*; BS: fed with the basal diet and 1 × 10^10^ CFU/g *B. subtilis.* Data are expressed as mean ± SD (*n* = 6 per group).

Figure 8A illustrates the results of the cat serum metabolomics sequencing. Supervised mode identification of OPLS-DA showed that the experimental groups clustered distinctly. As shown in Figure 8A,D, the addition of BL and BS led to a clear separation between these two groups and the CON group. This result indicates that the metabolic characteristics of the two groups differ significantly from those of the control group. The KEGG total compound classification statistics chart shows that the BL group consists primarily of peptides, hormones, and transmitters (Figure 8B), whereas the BS group consists primarily of carbohydrates and lipids (Figure 8E). As shown in the volcano plot, it presents the expression levels of metabolites that were differentially expressed after the addition of BL. In total, 191 metabolites were significantly upregulated and 43 were downregulated in the BL group relative to the control group (Figure 8C). Following the addition of BL, 87 metabolites exhibited significant upregulation and 76 showed significant downregulation (Figure 8F).

**Figure 9:**
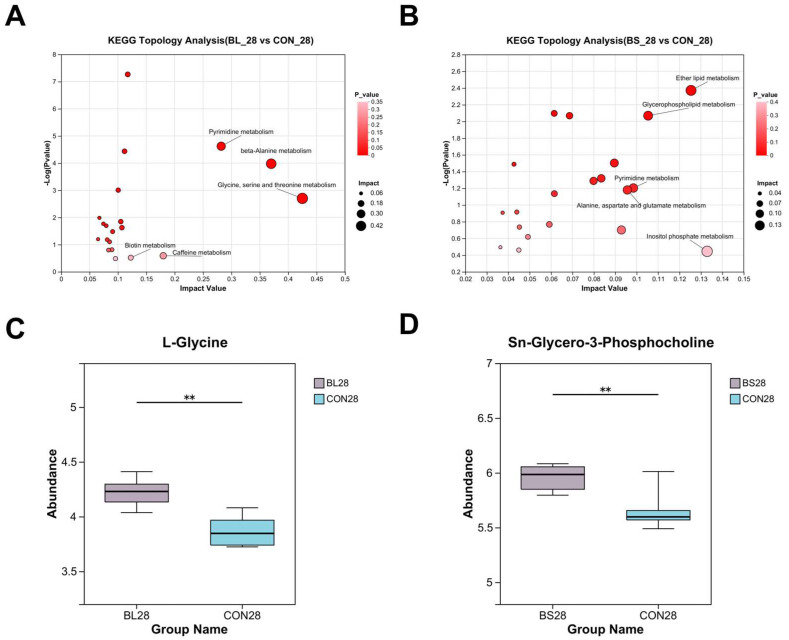
Effect of BL and BS on differential metabolites in cats. (**A**) KEGG Topology Analysis for BL group; (**B**) KEGG Topology Analysis for BS group (**C**) Differential metabolites in the BL group; (**D**) Differential metabolites in the BS group. CON: fed with basic diet; BL: fed with the basal diet and 1 × 10^10^ CFU/g *B. licheniformis*; BS: fed with the basal diet and 1 × 10^10^ CFU/g *B. subtilis.*, ** *p* ≤ 0.01. Data are expressed as mean ± SD (*n* = 6 per group).

The KEGG Topology Analysis of BL and BS Group Serum is displayed in Figure 9A,B. The results showed that the abundance of the synthesis pathways for Pyrimidine metabolism, β-Alanine metabolism, Glycine, serine and threonine metabolism, and Biotin metabolism increased in the BL group (Figure 9A). Furthermore, the BS group exhibited increased activity in the synthesis pathways of Ether Lipid metabolism, Pyrimidine metabolism, Alanine, aspartate and glutamate metabolism, Glycerophospholipid metabolism, and Inositol phosphoric acid metabolism (Figure 9B). Among the identified differential metabolites, the level of L-Glycine in the BL group was found to be significantly higher compared with that in the control group (Figure 9C; *p* < 0.05), while sn-Glycero-3-Phosphocholine was detected at a significantly higher level in the BS group (Figure 9D; *p* < 0.05).

## 4. Discussion

As cats become more popular as pets, greater attention is being paid to their health issues, such as diarrhea, obesity, and urinary problems. The gut is crucial for animal health, especially the microbes in it, which affect how nutrients are absorbed and metabolized [4]. Probiotics, which are beneficial live microorganisms, offer several advantages, including a high safety profile, non-toxicity, absence of residual accumulation, and minimal environmental impact [3]. The concentration of probiotics required for a clinical effect is often quoted as being at least 10^6^ CFU/mL in the small bowel and at least 10^8^ CFU/g in the colon [16]. Guo confirmed that a high-dose probiotic (*Bacillus* spp., *Bifidobacterium* spp., *Clostridium butyricum*, *Lactobacilli* spp., *Lactococcus* spp., *Leuconostoc cremoris*, *Saccharomyces* spp., or *Streptococcus* spp.) regimen demonstrates superior therapeutic efficacy over low-dose administration in managing antibiotic-associated diarrhea [17]. This finding aligns with the dose–effect threshold reported by Ouwehand [18]. Consequently, the probiotic dosage administered in this study was established at 1 × 10^10^ CFU/day, selected based on previous dose-response studies. Probiotic supplementation demonstrates therapeutic potential in enhancing intestinal homeostasis and reducing diarrheal incidence [19]. Torres-Henderson and colleagues found that the *Enterococcus faecium strain* SF68 reduced fecal scores and ameliorated diarrhea in their study population [20]. The supplementation of BL and BS in the present study significantly improved fecal scores in cats, which aligns with previous findings.

IgA represents the predominant immunoglobulin isotype across mucosal surfaces, serving as the primary immunological barrier against pathogenic microbial colonization and invasion. The present study has demonstrated that dietary supplementation with BL significantly enhances serum IgA levels in juvenile pigs [21]. IgM is a primary antibody generated during the initial immune response to antigen exposure. The substance exhibits potent bactericidal activity, robust complement system activation, and immunomodulatory functions [22]. IgG is the predominant antibody found in serum, being particularly prevalent in blood and other extracellular fluids [23]. The results showed that adding BL and BS to the diet of broilers significantly increased their serum IgA and IgM levels, which suggests that the broilers’ humoral immune capacity was enhanced [24]. Dong demonstrated that dietary supplementation with *Bacillus subtilis* BYS2 significantly increased serum IgG levels in broilers, with a 31.6% elevation compared to control-group counterparts [25]. Consistent with established literature, the current study demonstrated that BL and BS supplementation significantly elevated serum IgA and IgM concentrations in broilers. In contrast, BL administration failed to induce statistically significant increases in IgG levels, potentially attributable to the limited duration of the experimental intervention.

Serum antioxidant enzymes, including GSH-Px, T-AOC, SOD, and CAT, constitute critical elements of the endogenous antioxidant defense system. Acting in coordination, these enzymes mitigate oxidative harm through the neutralization of excess reactive oxygen species (ROS), a process that helps sustain cellular redox homeostasis [26]. SOD is an important endogenous antioxidant enzyme that acts as part of the body’s first line of defense against ROS [27]. Moreover, the glutathione system plays a pleiotropic role in defending cells against metabolic, oxidative, and metal stresses [28]. Lei observed that BS supplementation enhanced the antioxidant capacity of laying hens [29], and Bai revealed that *Bacillus subtilis* fmbJ (CGMCCN 0943) could increase the antioxidant capacity of broiler chickens by increasing GSH-Px and SOD activities in serum [30]. Consistent with previous research, we observed that BS increased GSH-Px, SOD, CAT, and T-AOC activities, and BL increased GSH-Px and T-AOC.

TNF-α is a pro-inflammatory cytokine that plays a central role in modulating innate immunity [31,32]. IFN-γ is crucial for maintaining tissue homeostasis, immune responses, and inflammatory reactions [33]. IL-2 is a crucial cytokine that regulates immune homeostasis and promotes T-cell activation, proliferation, and differentiation [34]. Dietary probiotic supplementation significantly reduced serum TNF-α concentrations in weaned piglets compared to the CON group, demonstrating an anti-inflammatory effect [35]. Research has revealed that the supplementation of *Bacillus licheniformis* MCC 2514 and *Bifidobacterium breve* NCIM 5671 downregulates the expression of pro-inflammatory cytokines while upregulating anti-inflammatory cytokines, including IL-4 [36]. Previous research conducted in our laboratory indicates that BL supplementation upregulates anti-inflammatory cytokine IL-4 and downregulates pro-inflammatory TNF-α levels [37]. In alignment with prior research, our study observed a reduction in pro-inflammatory cytokines, such as TNF-α and IFN-γ. Nevertheless, there was no substantial alteration in the concentrations of IL-4 and IL-2. This could be due to the inadequate dosage and duration of the experiment.

The gastrointestinal tract represents a major immunological organ, playing an essential role in host defense against pathogens. Probiotic colonization in the intestinal lumen modulates gut microbiota composition, thereby enhancing intestinal homeostasis and immune function [38]. D-LA and DAO serve as complementary biomarkers of intestinal barrier integrity, with their combined assessment providing a more robust evaluation of intestinal health status [39]. Yang demonstrated that *Bacillus subtilis* HH2 supplementation significantly reduced serum DAO activity following enterotoxigenic Escherichia coli (ETEC) challenge in beagles, concomitant with the amelioration of diarrheal symptoms [40]. Adding *Bifidobacterium lactis* (*B. lactis*) and *Lactobacillus plantarum* significantly improved intestinal barrier function. Plasma D-LA concentration decreased by 30.38%, as did DAO concentration (by 22.68%) [41]. The current study demonstrated that both BL and BS supplementation significantly reduced serum D-LA concentrations and DAO activity compared to control groups, consistent with previous reports of their intestinal barrier-protective effects.

Specifically, Fusobacteria, Bacteroidetes, and Firmicutes were identified as the dominant and most prevalent bacterial phyla in the fecal microbiota of both dogs and cats [42,43]. The present study identified that the dominant phyla within the intestinal microbiota are Bacillota, Actinomycetota, Bacteroidota, and Pseudomonadota, which differ from previous reports. This discrepancy may be due to host-specific factors, such as breed (predominantly Ragdoll cats) and age (>1 year). The commensal bacterium *Faecalibacterium prausnitzii* plays a key role in IBD pathogenesis, and Butyrate mediates the anti-inflammatory effects of *Faecalibacterium prausnitzii* in intestinal epithelial cells through Dact3 [44]. Previous studies have suggested that Butyrate exerts potent effects on a variety of colonic mucosal functions, such as inhibition of inflammation and carcinogenesis, reinforcing various components of the colonic defense barrier and decreasing oxidative stress [45]. Given that *Faecalibacterium* is a major butyrate producer, it could increase intestinal butyrate levels by modulating beneficial microbiota. This would contribute to improved intestinal barrier function and overall gut homeostasis. Microbial compositional analysis revealed that the BL increased the relative abundance of *Faecalibacterium*, and the BL and BS increased the relative abundance of *Bacillus*, while the *Mogibacterium* relative abundance was decreased. Prior research has indicated that *Bacillus* hs a protective effect and performs functions that are important for sustaining gut health [46]. Previous studies have established that the relative abundance of *Mogibacterium* within the microbiome of patients with colorectal cancer (CRC) increases progressively from mucosal-invasive carcinoma to advanced stages [47]. M Oba demonstrated that *B. subtilis* ATCC PTA-122264 significantly reduced fecal abundance of potential pathogens, including *Streptococcus spp. Escherichia coli, and Cyanobacteria*, suggesting its antimicrobial efficacy in modulating gut microbiota composition [48]. Consistent with previous findings, BL and BS improve the health of the intestinal tract by increasing the levels of beneficial bacterial taxa such as *Faecalibacterium* and *Bacillus*, and decreasing the levels of detrimental bacteria, including *Mogibacterium*. Within this study, the BL group exhibited a total of 3777 differential metabolites relative to the control group. The primary annotated metabolic pathways included Pyrimidine metabolism, beta-Alanine metabolism, and Glycine, serine, and threonine metabolism. Notably, the level of the metabolite L-Glycine in the BL group was significantly upregulated. Similarly, the BS group showed 3848 differential metabolites, with major pathways enriched in Lipid metabolism, Pyrimidine metabolism, Glycerophospholipid metabolism, and Alanine, aspartate, and glutamate metabolism. Likewise, the level of sn-Glycero-3-Phosphocholine in the ether lipid metabolism pathway of the BS group showed an upregulated trend. The distinct metabolic reprogramming induced by BL (amino acid metabolism emphasis) versus BS (Lipid metabolism focus) suggests strain-specific bioactivity. Sn-Glycero-3-Phosphocholine serves as a critical biosynthetic precursor for phosphatidylcholine (PC). Studies have underscored the importance of PC metabolism, in which the balance between its anabolic and catabolic processes is essential for preserving the structural integrity of cellular membranes, supporting hepatic function, and ensuring proper neurological activity [49]. Through 16S rRNA gene sequencing analysis, both BL and BS supplementation increased the abundance of beneficial bacteria, including *Faecalibacterium*. As a butyrate producer, *Faecalibacterium* contributes to the suppression of inflammation and reinforcement of the intestinal barrier. Concurrently, sn-Glycero-3-Phosphocholine, a precursor for phospholipid membrane synthesis, provides essential substrates for the regeneration of intestinal cells. Together, these effects create a low-inflammatory microenvironment with enhanced barrier integrity, which in turn facilitates the colonization and expansion of beneficial bacteria. These findings are consistent with the observed attenuation of inflammatory markers and the promotion of a beneficial microbiota composition. Research has demonstrated that L-Glycine exhibits significant potential in mitigating inflammatory responses, modulating immune functions, and preserving cellular viability [50,51]. These results align with those of prior studies, confirming that BL supplementation effectively lowers serum TNF-α levels and mitigates inflammatory responses. Glycine is a constituent of significant proportions in the proteins of both gram-positive and gram-negative gut bacteria [52]. This result suggests that glycine has a crucial function in fostering the optimal proliferation of the intestinal microbiota [53]. Our research revealed that elevated serum L-glycine levels, alongside an increased abundance of *Faecalibacterium* and *Bacillus*, may be the result of L-Glycine’s ability to maintain intestinal homeostasis, encourage the growth of beneficial bacteria, and strengthen the intestinal functional barrier.

## 5. Conclusions

In conclusion, dietary supplementation of BL and BS can improve the immune ability of cats by increasing the content of IgA and IgM in serum, and it has significant antioxidant capacity and potent inflammatory protection. Additionally, the supplementation of BL and BS can elevate the abundance of beneficial bacterial taxa while lowering the abundance of detrimental bacteria. This strategy improves the health of the intestinal tract by adjusting the structure of the gut microbiota. Analytical findings indicated that the differentially abundant metabolites in the BL group were primarily tied to amino acid metabolism pathways, whereas those in the BS group were mainly related to lipid metabolism. Despite these findings, this study has limitations. The sample was restricted to Ragdoll cats, warranting future investigations across diverse breeds to comprehensively assess the health impacts of BL and BS. Additionally, the 35-day experimental period captured only short-term physiological responses; extended studies are needed to evaluate long-term effects. Collectively, these results suggest that BL and BS possess considerable potential for applications in intestinal health maintenance.

## Figures and Tables

**Table 1:** Fecal scoring criteria ^1^.

Fecal State	Score
Hard and dry, particles are discharged, friable in lumps	5
Feces’ surface shows a segmented texture and leaves little residue when picked up	4
The surface of the feces is moist and log-like, and picking up will leave residue	3
Feces are piled, not fully liquid, hard to pick up, and leave residues when lifted	2
Liquid and difficult to pick up	1

^1^ Fecal Score as proposed by Carciofi [15].

**Table 2:** Effects of BL and BS on ADFI and body weights ^1^.

Item	Groups ^2^			*p*-Value
	CON	BL	BS	
ADFI, g/d	51.73 ± 15.35	53.51 ± 16.60	51.05 ± 13.09	0.318
D1 Body weight, kg	3.40 ± 0.12	3.68 ± 0.93	3.71 ± 0.70	0.783
D7 Body weight, kg	3.40 ± 0.15	3.70 ± 0.87	3.69 ± 0.76	0.792
D14 Body weight, kg	3.45 ± 0.15	3.68 ± 0.93	3.71 ± 0.70	0.841
D21 Body weight, kg	3.47 ± 0.18	3.77 ± 0.81	3.76 ± 0.68	0.736
D28 Body weight, kg	3.49 ± 0.18	3.74 ± 0.94	3.71 ± 0.56	0.837

^1^ Data are expressed as mean ± SD (*n* = 6 per group). ^2^ CON: fed with basic diet; BL: fed with the basal diet and 1 × 10^10^ CFU/g *B. licheniformis*; BS: fed with the basal diet and 1 × 10^10^ CFU/g *B. subtilis.*

**Table 3:** Effects of BL and BS on fecal scores in cats ^1^.

Item	Groups ^2^			*p*-Value
	CON	BL	BS	
D1	3.33 ± 1.03	3.67 ± 0.52	3.83 ± 0.75	0.554
D2	3.33 ± 1.03	4.17 ± 0.41	4.33 ± 0.82	0.098
D3	3.50 ± 0.84 b	4.17 ± 0.41 ab	4.50 ± 0.55 a	0.041
D4	3.33 ± 0.82 b	4.50 ± 0.55 a	4.17 ± 0.75 ab	0.035
D5	3.33 ± 0.82 b	4.33 ± 0.52 a	4.33 ± 0.82 a	0.048
D6	3.50 ± 0.55	3.83 ± 0.75	4.33 ± 0.82	0.161
D7	3.33 ± 0.82 b	3.33 ± 0.52 b	4.67 ± 0.52 a	0.003
D14	3.33 ± 0.82 b	3.50 ± 0.55 b	4.67 ± 0.82 a	0.013
D21	3.50 ± 0.55 b	4.00 ± 0.89 ab	4.67 ± 0.82 a	0.057
D28	4.00 ± 0.63	4.00 ± 0.63	4.50 ± 0.84	0.391

a, b: means within the same row that have different superscripts are significantly different (*p* < 0.05). ^1^ Data are expressed as mean ± SD (*n* = 6 per group). ^2^ CON: fed with basic diet; BL: fed with the basal diet and 1 × 10^10^ CFU/g *B. licheniformis*; BS: fed with the basal diet and 1 × 10^10^ CFU/g *B. subtilis.*

## Data Availability

The DNA sequences of this article were deposited in the National Center for Biotechnology Information (NCBI) Sequence Read Archive (SRA) repository under accession number PRJNA1332594.

## Abbreviations

The following abbreviations are used in this article:

BL	Bacillus licheniformis
BS	Bacillus subtilis
B. licheniformis	Bacillus licheniformis
B. subtilis	Bacillus subtilis
IBD	Inflammatory bowel disease
AOPP	Advanced oxidation protein products
d-ROMS	Diacron-reactive oxygen metabolites
TBARS	Thiobarbituric acid reactive substances
LPS	Lipopolysaccharide
SOD	Superoxide dismutase
T-AOC	Total antioxidant capacity
GSH-Px	Glutathione peroxidase
CAT	Catalase
DLA	D-lactic acid
TNF-α	Tumor necrosis factor-α
IFN-γ	Interferon-γ
IL	Interleukin
Ig	Immunoglobulin
ELISA	Enzyme-linked immunosorbent assay
PCR	Polymerase chain reaction
PCoA	Principal Coordinate Analysis
OPLS-DA	Orthogonal projections to latent structures-discriminant analysis
VIP	variable importance in projection
KEGG	Kyoto Encyclopedia of Genes and Genomes
ADFI	Average daily feed intake
ASVs	Amplicon sequence variants
B. lactis	Bifidobacterium lactis

## References

[1] Markowiak P., Śliżewska K. (2017). Effects of probiotics, prebiotics, and synbiotics on human health. Nutrients.

[2] Alessandri G., Argentini C., Milani C., Turroni F., Ossiprandi M.C., Sinderen D.V., Ventura M. (2020). Catching a glimpse of the bacterial gut community of companion animals: A canine and feline perspective. Microb. Biotechnol..

[3] Zha M., Zhu S., Chen Y. (2024). Probiotics and cat health: A review of progress and prospects. Microorganisms.

[4] Lee D., Goh T.W., Kang M.G., Choi H.J., Yeo S.Y., Yang J., Huh C.S., Kim Y.Y., Kim Y. (2022). Perspectives and advances in probiotics and the gut microbiome in companion animals. J. Anim. Sci. Technol..

[5] Jergens A.E. (2012). Feline idiopathic inflammatory bowel disease. J. Feline Med. Surg..

[6] Tizard I.R., Jones S.W. (2018). The microbiota regulates immunity and immunologic diseases in dogs and cats. Vet. Clin. N. Am. Small Anim. Pract..

[7] Li Y., Ali I., Lei Z., Li Y., Yang M., Yang C., Li L. (2023). Effect of a multistrain probiotic on feline gut health through the fecal microbiota and its metabolite SCFAs. Metabolites.

[8] Mohammed E.A.H., Ahmed A.E.M., Kovács B., Pál K. (2025). The Significance of Probiotics in Aquaculture: A Review of Research Trend and Latest Scientific Findings. Antibiotics.

[9] Gresse R., Chaucheyras-Durand F., Fleury M.A., Van de Wiele T., Forano E., Blanquet-Diot S. (2017). Gut Microbiota Dysbiosis in Postweaning Piglets: Understanding the Keys to Health. Trends Microbiol..

[10] Feng S., Meng C., Hao Z., Liu H. (2022). *Bacillus licheniformis* reshapes the gut microbiota to alleviate the subhealth. Nutrients.

[11] Setlow P. (2014). Spore resistance properties. Microbiol. Spectr..

[12] Allenspach K., Sung C.-H., Ceron J.J., Peres Rubio C., Bourgois-Mochel A., Suchodolski J.S., Yuan L., Kundu D., Colom Comas J., Rea K. (2023). Effect of the probiotic bacillus subtilis DE-CA9TM on fecal scores, serum oxidative stress markers and fecal and serum metabolome in healthy dogs. Vet. Sci..

[13] Félix A.P., Netto M.V.T., Murakami F.Y., de Brito C.B.M., de Oliveira S.G., Maiorka A. (2010). Digestibility and fecal characteristics of dogs fed with Bacillus subtilis in diet. Cienc. Rural.

[14] Yu X., Dai Z., Cao G., Cui Z., Zhang R., Xu Y., Wu Y., Yang C. (2023). Protective effects of *Bacillus licheniformis* on growth performance, gut barrier functions, immunity and serum metabolome in lipopolysaccharide-challenged weaned piglets. Front. Immunol..

[15] Carciofi A.C., de-Oliveira L.D., Valério A.G., Borges L.L., de Carvalho F.M., Brunetto M.A., Vasconcellos R.S. (2009). Comparison of micronized whole soybeans to common protein sources in dry dog and cat diets. Anim. Feed. Sci. Technol..

[16] Minelli E.B., Benini A. (2008). Relationship between number of bacteria and their probiotic effects. Microb. Ecol. Health Dis..

[17] Guo Q., Goldenberg J.Z., Humphrey C., El Dib R., Johnston B.C. (2019). Probiotics for the prevention of pediatric antibiotic-associated diarrhea. Cochrane Database Syst. Rev..

[18] Ouwehand A.C. (2017). A review of dose-responses of probiotics in human studies. Benef. Microbes.

[19] Xu H., Zhao F., Hou Q., Huang W., Liu Y., Zhang H., Sun Z. (2019). Metagenomic analysis revealed beneficial effects of probiotics in improving the composition and function of the gut microbiota in dogs with diarrhoea. Food Funct..

[20] Torres-Henderson C., Summers S., Suchodolski J., Lappin M.R. (2017). Effect of enterococcus faecium strain SF68 on gastrointestinal signs and fecal microbiome in cats administered amoxicillin-clavulanate. Top. Companion Anim. Med..

[21] Yu X., Cui Z., Qin S., Zhang R., Wu Y., Liu J., Yang C. (2022). Effects of *Bacillus licheniformis* on growth performance, diarrhea incidence, antioxidant capacity, immune function, and fecal microflora in weaned piglets. Animals.

[22] Franklin E.C., Frangione B. (1969). Immunoglobulins. Annu. Rev. Med..

[23] Lux A., Aschermann S., Biburger M., Nimmerjahn F. (2010). The pro and anti-Inflammatory activities of immunoglobulin G. Ann. Rheum. Dis..

[24] Xu Y., Yu Y., Shen Y., Li Q., Lan J., Wu Y., Zhang R., Cao G., Yang C. (2021). Effects of Bacillus subtilis and *Bacillus licheniformis* on growth performance, immunity, short chain fatty acid production, antioxidant capacity, and cecal microflora in broilers. Poult. Sci..

[25] Dong Y., Li R., Liu Y., Ma L., Zha J., Qiao X., Chai T., Wu B. (2020). Benefit of dietary supplementation with Bacillus subtilis BYS2 on growth performance, immune response, and disease resistance of broilers. Probiotics Antimicrob. Proteins.

[26] Feng J., Cai Z., Chen Y., Zhu H., Chang X., Wang X., Liu Z., Zhang J., Nie G. (2020). Effects of an exopolysaccharide from lactococcus lactis Z-2 on innate immune response, antioxidant activity, and disease resistance against aeromonas hydrophila in *Cyprinus carpio* L. Fish Shellfish Immunol..

[27] Ighodaro O.M., Akinloye O.A. (2018). First line defence antioxidants-superoxide dismutase (SOD), catalase (CAT) and glutathione peroxidase (GPX): Their fundamental role in the entire antioxidant defence grid. Alex. J. Med..

[28] Cassier-Chauvat C., Marceau F., Farci S., Ouchane S., Chauvat F. (2023). The glutathione system: A journey from cyanobacteria to higher eukaryotes. Antioxidants.

[29] Lei K., Li Y.L., Yu D.Y., Rajput I.R., Li W.F. (2013). Influence of dietary inclusion of *Bacillus licheniformis* on laying performance, egg quality, antioxidant enzyme activities, and intestinal barrier function of laying hens. Poult. Sci..

[30] Bai K., Huang Q., Zhang J., He J., Zhang L., Wang T. (2017). Supplemental Effects of probiotic Bacillus subtilis fmbJ on growth performance, antioxidant capacity, and meat quality of broiler chickens. Poult. Sci..

[31] Montgomery S.L., Bowers W.J. (2012). Tumor Necrosis Factor-Alpha and the Roles It plays in homeostatic and degenerative processes within the central nervous system. J. Neuroimmune Pharmacol..

[32] Yao Q., He L., Bao C., Yan X., Ao J. (2024). The role of TNF-α in osteoporosis, bone repair and inflammatory bone diseases: A review. Tissue Cell.

[33] Ivashkiv L.B. (2018). IFNγ: Signalling, epigenetics and roles in immunity, metabolism, disease and cancer immunotherapy. Nat. Rev. Immunol..

[34] Lan R.Y., Selmi C., Gershwin M.E. (2008). The regulatory, inflammatory, and T cell programming roles of interleukin-2 (IL-2). J. Autoimmun..

[35] Cao G., Tao F., Hu Y., Li Z., Zhang Y., Deng B., Zhan X. (2019). Positive effects of a clostridium butyricum-based compound probiotic on growth performance, immune responses, intestinal morphology, hypothalamic neurotransmitters, and colonic microbiota in weaned piglets. Food Funct..

[36] HS R., Halami P.M. (2023). The combined effect of potential probiotic *Bacillus licheniformis* MCC 2514 and bifidobacterium breve NCIM 5671 towards anti-inflammatory activity on HT-29 cell lines. Probiotics Antimicrob. Proteins.

[37] Zhong Y., Zhang M., Xu H., Yu X., Hu Y., Xu Y., Xiao X., Yang C. (2025). *Bacillus licheniformis* alleviates clostridium perfringens-induced intestinal injury in mice model by modulating inflammation, apoptosis, and cecal microbial-metabolic responses. Animals.

[38] Hoffmann A., Kleniewska P., Pawliczak R. (2021). Antioxidative activity of probiotics. Arch. Med. Sci..

[39] Liang S.-J., Wang X.-Q. (2023). Deoxynivalenol induces intestinal injury: Insights from oxidative stress and intestinal stem cells. Environ. Sci. Pollut. Res. Int..

[40] Yang J., Zhang X., Zhou Z., Li C., Luo R., Liu H., Fu H., Zhong Z., Shen L., Cao S. (2023). Protective effects of Bacillus subtilis HH2 against oral enterotoxigenic escherichia coli in beagles. Vet. Sci..

[41] Wang W., Dong H., Chang X., Chen Q., Wang L., Chen S., Chen L., Wang R., Ge S., Wang P. (2024). Bifidobacterium lactis and lactobacillus plantarum enhance immune function and antioxidant capacity in cats through modulation of the gut microbiota. Antioxidants.

[42] Handl S., Dowd S.E., Garcia-Mazcorro J.F., Steiner J.M., Suchodolski J.S. (2011). Massive parallel 16S rRNA gene pyrosequencing reveals highly diverse fecal bacterial and fungal communities in healthy dogs and cats. FEMS Microbiol. Ecol..

[43] Ritchie L.E., Burke K.F., Garcia-Mazcorro J.F., Steiner J.M., Suchodolski J.S. (2010). Characterization of fecal microbiota in cats using universal 16S rRNA gene and group-specific primers for *lactobacillus* and *bifidobacterium* spp. Vet. Microbiol..

[44] Lenoir M., Martín R., Torres-Maravilla E., Chadi S., González-Dávila P., Sokol H., Langella P., Chain F., Bermúdez-Humarán L.G. (2020). Butyrate mediates anti-inflammatory effects of faecalibacterium prausnitzii in intestinal fpithelial cells through Dact3. Gut Microbes.

[45] Hamer H.M., Jonkers D., Venema K., Vanhoutvin S., Troost F.J., Brummer R.-J. (2008). Review Article: The role of butyrate on colonic function. Aliment. Pharmacol. Ther..

[46] Wang F., Mei X., Wang Q., Zhao P., Zhou Y., Tang L., Wang B., Xu S., Li X., Jin Q. (2023). Compound Bacillus alleviates diarrhea by regulating gut microbes, metabolites, and inflammatory responses in pet cats. Anim. Microbiome.

[47] Xu Y.-J., He Y., Chen C., Shi J., He M., Liu Y., Zhang Y., Liu Y., Zhang Y. (2024). Multiomics analysis revealed colorectal cancer pathogenesis. J. Proteome Res..

[48] Oba P.M., Swanson O.R., Kang Y., Mioto J.C., Menton J.F., Vinay E., Millette M., Kelly M.R., Swanson K.S. (2025). Effects of Bacillus subtilis ATCC PTA-122264 on apparent total tract macronutrient digestibility and fecal characteristics, metabolites, and microbiota of healthy adult dogs. J. Anim. Sci..

[49] van der Veen J.N., Kennelly J.P., Wan S., Vance J.E., Vance D.E., Jacobs R.L. (2017). The critical role of phosphatidylcholine and phosphatidylethanolamine metabolism in health and disease. Biochim. Biophys. Acta (BBA)—Biomembr..

[50] Zhong Z., Wheeler M.D., Li X., Froh M., Schemmer P., Yin M., Bunzendaul H., Bradford B., Lemasters J.J. (2003). L-Glycine: A novel antiinflammatory, immunomodulatory, and cytoprotective agent. Curr. Opin. Clin. Nutr. Metab. Care.

[51] Petrat F., Boengler K., Schulz R., de Groot H. (2012). Glycine, a simple physiological compound protecting by yet puzzling mechanism(s) against ischaemia-reperfusion injury: Current knowledge. Br. J. Pharmacol..

[52] Dai Z.-L. (2011). Amino acid metabolism in intestinal bacteria: Links between gut ecology and host health. Front. Biosci..

[53] Alves A., Bassot A., Bulteau A.-L., Pirola L., Morio B. (2019). Glycine metabolism and its alterations in obesity and metabolic diseases. Nutrients.

